# Noncoding RNAs in Nuclear Condensates and RNA Processing in Skeletal Muscle

**DOI:** 10.3390/ijms27219713

**Published:** 2026-10-29

**Authors:** Thanh Huu Phan Ngo, Wan Lee

**Affiliations:** 1 Department of Biochemistry, College of Medicine, Dongguk University, 123 Dongdae-ro, Gyeongju 38066, Republic of Korea; thngo139@gmail.com; 2 Medical Research Institute, College of Medicine, Dongguk University, 123 Dongdae-ro, Gyeongju 38066, Republic of Korea

**Keywords:** noncoding RNA, skeletal muscle, nuclear compartment, RNA processing, myotonic dystrophy, RNA foci

## Abstract

Spatial organization in the nucleus is an active layer of RNA regulation in skeletal muscle, yet localization is often interpreted more strongly than the evidence permits. This critical review compares physiological nuclear RNA compartments with pathological repeat-RNA assemblies using a graded evidence framework. NEAT1-associated paraspeckles provide the clearest muscle-linked example of an RNA-marked nuclear body, although isoform-specific architectural causality remains untested in muscle. MALAT1 regulates speckle-associated splicing-factor and chromatin states but is dispensable for gross speckle formation, and muscle studies report opposing differentiation phenotypes. Nucleolar FRG2 transcripts in facioscapulohumeral muscular dystrophy connect spatial RNA organization to rDNA transcription and translational capacity. Expanded DMPK CUG and CNBP CCUG RNAs in myotonic dystrophy sequester processing factors and drive spliceopathy, although calibrated imaging in patient-derived DM1 myotubes indicates that most apparent foci are individual retained transcripts rather than bulk condensates. The framework distinguishes spatial association, molecular participation, functional necessity, in vivo relevance, and therapeutic target engagement. It also maps interventions to toxic-RNA abundance, RNA–protein binding, repeat-locus transcription, downstream splicing, or compartment state. Together, this evidence-based synthesis identifies which nuclear RNA mechanisms are established in muscle, which remain extrapolated from non-muscle systems, and which experiments are required before therapeutic modulation can be interpreted mechanistically.

## 1. Introduction

Skeletal muscle differentiation requires coordinated post-transcriptional regulation [1], while nuclear architecture spatially organizes gene-regulatory and RNA-processing activities [2]. A proliferating myoblast and a post-mitotic myotube therefore differ not only in the abundance of individual RNAs and proteins, but also in where these molecules are concentrated and which interactions become possible. Nuclear bodies can retain selected transcripts, partition RNA-binding proteins, and support RNA processing [2]. These spatial effects are especially relevant to muscle because alternative splicing, transcriptional competence, and ribosome production must be remodeled over a short developmental interval and then maintained in a large, multinucleated cell.

Long noncoding RNAs can participate in this organization in several distinct ways. An RNA may be necessary for assembly of a nuclear body, regulate the activity of proteins within an existing compartment, transiently enter a compartment without determining its structure, or accumulate because a pathological transcript cannot be exported or degraded. These roles are not interchangeable. The distinction is visible in three muscle-relevant systems. NEAT1 marks paraspeckles and has a defined architectural long isoform [3,4]. MALAT1 is concentrated at nuclear speckles but is dispensable for their gross structural integrity [5,6]. Expanded DMPK or CNBP repeat RNAs form nuclear foci in myotonic dystrophy and reduce the available pool of splicing regulators [7,8]. The similarities are spatial; the drivers, dynamics, and biological consequences differ.

The term biomolecular condensate adds a further evidentiary requirement. Microscopic concentration, round morphology, or RNA fluorescence in situ hybridization (FISH) puncta do not by themselves establish phase separation. A condensate claim is stronger when assembly depends on defined multivalent interactions and when the structure shows dynamic exchange, concentration buffering, reversibility, fusion, or another material-property measurement [9]. Paraspeckles satisfy several of these criteria in non-muscle experimental systems, including domain-resolved assembly and phase-separation assays [10,11,12]. Whether the same properties determine a particular muscle phenotype must still be tested in the relevant muscle state.

This review addresses four connected questions. Which nuclear noncoding RNAs are linked to defined compartments or processing reactions in skeletal muscle? Which experiments distinguish architectural causality from expression change or colocalization? How do physiological compartments differ from repeat-RNA assemblies that drive spliceopathy? Finally, which therapeutic interventions have demonstrated target engagement at the relevant causal layer? Throughout the manuscript, “nuclear body” or “compartment” denotes a spatially organized domain supported by localization and accepted component markers. “RNA focus” denotes a discrete RNA signal without an implied material state. “Condensate” is reserved for assemblies supported by evidence of multivalent interactions and relevant material behavior.

The scope is intentionally selective. Cytoplasmic competing-endogenous-RNA networks, extracellular-vesicle RNAs, and expression-only biomarker studies are outside the central argument. Chromatin-associated lncRNAs are included only when physical localization or interaction is connected to a defined nuclear process. Non-muscle studies establish experimental principles but are labeled as extrapolations. This boundary keeps the review focused on spatial nuclear mechanisms and prevents a broad catalog of muscle lncRNAs from obscuring the evidence needed to assign causality. Literature identification, scope, and source-verification procedures are provided in the Supplementary Materials (Supplementary Methods S1). Search concept blocks and publication-status checks are provided in Tables S1 and S2, respectively.

## 2. Conceptual and Evidentiary Framework

### 2.1. Operational Definitions and Evidence Levels

A nuclear compartment is a spatially organized domain demonstrated by imaging or proximity-based measurements. A nuclear body is a recognized structure with accepted components, such as a paraspeckle, nuclear speckle, or nucleolus. An RNA focus is an operational imaging term: RNA signal is concentrated in a discrete region, without a claim about material state. A biomolecular condensate is a mechanistic interpretation that requires evidence for assembly through multivalent molecular interactions and for relevant material behavior. An architectural RNA is more restrictive still. Its depletion should impair assembly or maintenance, and an isoform- or domain-specific rescue should restore the structure when feasible.

These definitions separate the measured observable from the inferred biological quantity. RNA FISH measures spatial signal and, depending on calibration, molecule number. It does not establish direct binding or phase behavior. Biochemical fractionation measures recovery in an operational fraction, not immobility in the intact nucleus. Co-immunoprecipitation supports membership in the same biochemical complex, but not necessarily direct contact. Changes in exon inclusion after RNA depletion establish a functional relationship; they do not identify whether altered speckle organization, transcription kinetics, splicing-factor phosphorylation, or an indirect stress response is causal. Each inference therefore requires its own control.

Paraspeckles illustrate why isoform identity matters. Alternative 3′-end processing produces short NEAT1_1 and long NEAT1_2 transcripts, and HNRNPK-dependent production of NEAT1_2 initiates paraspeckle construction [3]. Genome editing showed that a substantial fraction of NEAT1_1 can localize outside paraspeckles, while NEAT1_1-specific loss produced limited phenotypes in the systems examined [13,14]. Semi-extractability is another useful property of several architectural nuclear RNAs, but it is a biochemical signature and is not sufficient proof of an architectural role [15]. A study reporting “NEAT1” without resolving isoform or assembly state may therefore establish a NEAT1-dependent phenotype without establishing a paraspeckle-dependent mechanism.

Nuclear speckles impose a different interpretive boundary. Super-resolution imaging supports a multilayer organization of speckle proteins and RNA [16]. MALAT1 is enriched around these domains and can alter the distribution and phosphorylation of serine/arginine-rich splicing factors in cultured human cells [5]. Yet three independent mouse loss-of-function studies found normal viability and no requirement for MALAT1 in gross speckle formation, with context-dependent effects on neighboring transcription or molecular phenotypes [6,17,18]. The evidence supports a regulatory, concentration-dependent role more strongly than an obligatory architectural role.

The evidence ladder used here assigns the permitted interpretation and does not rank a paper globally. E0 denotes expression association. E1 adds nuclear enrichment or colocalization. E2 establishes physical interaction or maps components. E3 shows that perturbation changes assembly or RNA processing. E4 adds rescue, domain mapping, epistasis, or an orthogonal perturbation. E5 links the mechanism to an in vivo or human phenotype. E6 requires target engagement together with molecular and phenotypic rescue. A single study can occupy different tiers for different claims. For example, a study may provide E5 evidence that an RNA affects muscle regeneration but only E1 evidence that the effect occurs through a named nuclear body.

**Figure 1:**
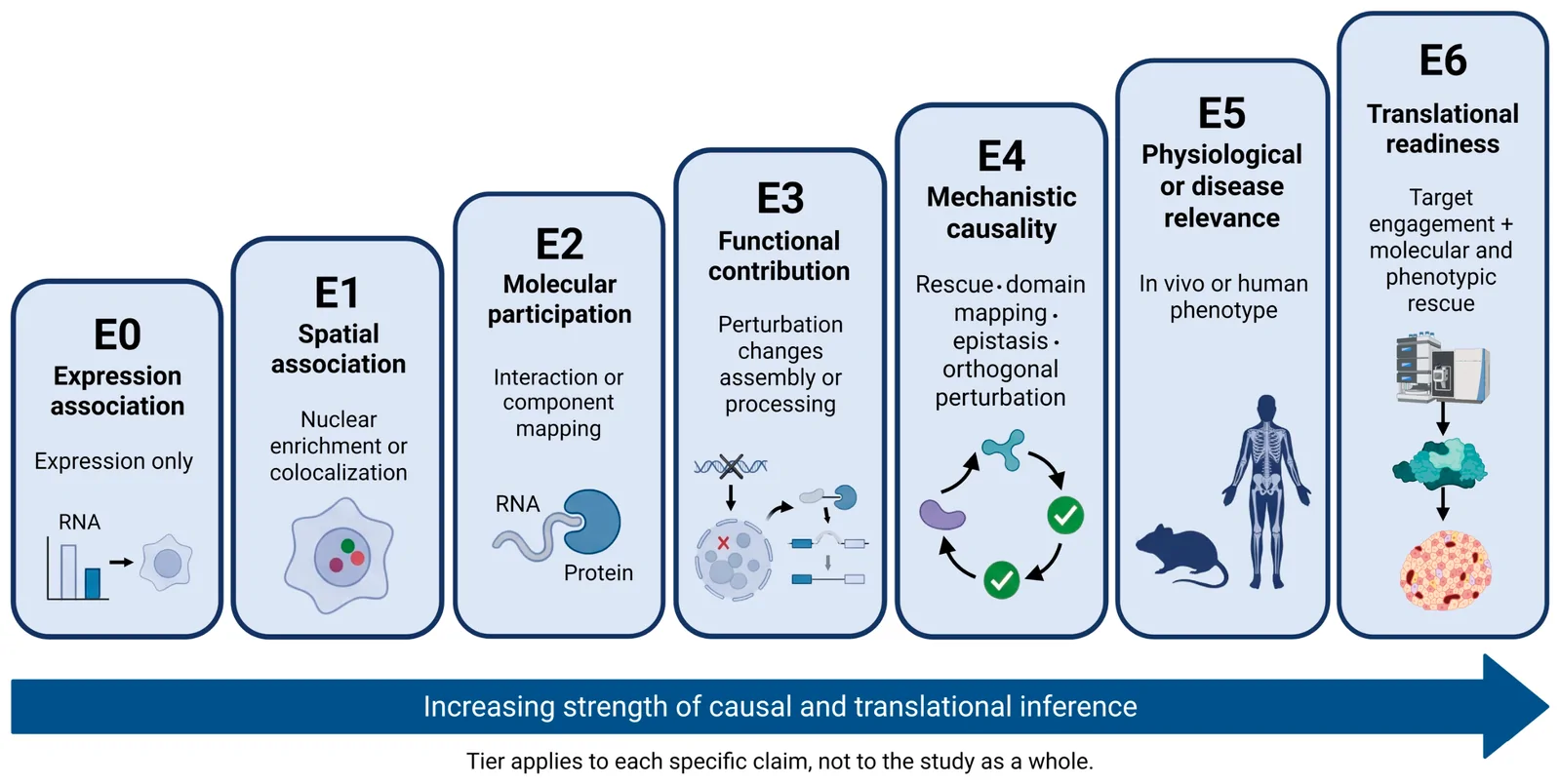
Claim-specific evidence ladder for nuclear RNA mechanisms in skeletal muscle. E0, expression association; E1, nuclear enrichment or colocalization; E2, physical interaction or component mapping; E3, a perturbation-dependent change in assembly or RNA processing; E4, mechanistic reinforcement by rescue, domain mapping, epistasis, or an orthogonal perturbation; E5, linkage to an in vivo or human phenotype; and E6, target engagement together with molecular and phenotypic rescue. The horizontal progression indicates increasing strength of causal and translational inference. Each tier applies to an individual claim rather than serving as a global quality score for a study. Created in BioRender. Lee, W. (2026) https://BioRender.com/i1l53l2.

Figure 1 provides a claim-specific summary of the E0–E6 ladder. Movement to a higher tier requires additional evidence and does not convert the tier into a global score for the study.

Table 1 applies the ladder to the muscle-relevant modules and identifies the strongest interpretation supported for each claim.

The ladder also constrains language. E1 supports “localizes to” or “is associated with,” not “scaffolds.” E2 supports participation in a molecular complex, not necessity. E3 supports a functional contribution within the tested perturbation and model. E4 strengthens causal assignment by showing that the phenotype tracks with a mapped RNA element, partner, or rescue construct. E5 is required before a cellular mechanism is presented as muscle physiology or disease biology. E6 is reserved for interventions that reach the intended target and correct a downstream molecular defect together with a relevant phenotype.

### 2.2. Physiological Compartments and Pathological RNA Assemblies

Physiological nuclear bodies are maintained by regulated synthesis, turnover, and exchange of their components. Their assembly can be coupled to differentiation or stress without being pathogenic. Repeat-expansion RNAs are produced from mutant alleles, retained for abnormal durations, and present repeated binding motifs that distort the availability of RNA-binding proteins. Both categories can concentrate RNA and protein, but the selective pressures and consequences are different. The comparison is useful only when it preserves these differences.

Table 2 makes this comparison explicit by aligning the RNA driver or resident, associated proteins, assembly evidence, processing output, and muscle-specific evidence boundary for each system.

This framework avoids two common errors. First, loss of a visible focus after treatment does not necessarily prove that dissolution of a condensate caused the biological rescue; the treatment may simply reduce RNA abundance. Second, a phenotype after depletion of a compartment-associated lncRNA does not prove that changing compartment architecture caused the phenotype. The decisive experiment measures RNA abundance, assembly state, molecular processing, and muscle output in the same perturbation series and includes a rescue that separates these variables.

Assembly and function should therefore be represented as separable causal nodes. RNA abundance can affect assembly, but it can also alter transcription, chromatin occupancy, or protein binding without changing a visible body. Assembly can affect processing, yet processing can also change because the same RNA or protein acts in the surrounding nucleoplasm. A muscle phenotype may arise from either route or from a change in cell-state composition. Factorial perturbations are more informative than a single knockdown: one intervention changes RNA abundance, a second changes assembly competence while preserving abundance, and a third restores the proposed downstream processing event. Concordant rescue across these levels supports the complete path. Divergent rescue identifies where the proposed mechanism fails.

## 3. NEAT1 and Paraspeckles in Myogenic State Transitions

Among the systems reviewed here, NEAT1 is the strongest muscle-linked candidate for an architectural nuclear RNA, but direct muscle studies currently separate into evidence for paraspeckle association and evidence for NEAT1-dependent myogenic regulation; few experiments connect both in the same system.

NEAT1 and MALAT1 are broadly, but not uniformly, expressed across human tissues and are not restricted to skeletal muscle. GTEx detects both transcripts in skeletal muscle and many other tissues [37]. Neither transcript is skeletal-muscle selective. MALAT1 is abundant across many tissues, whereas NEAT1 abundance and isoform balance vary across tissues and cell states [3,13,14]. Bulk RNA levels cannot be read as nuclear-body abundance because tissue composition, RNA extraction, and isoform resolution influence the measurement. Their relevance to muscle therefore depends on state-specific regulation and causal function, not muscle-selective expression.

### 3.1. Differentiation-Dependent Paraspeckle Association in C2C12 Cells

The foundational muscle study identified the then-named Men ε/β transcripts as differentiation-regulated, nuclear-retained RNAs and later recognized them as NEAT1 isoforms [4]. During C2C12 differentiation, Men ε/β transcript abundance increased, and RNA FISH showed larger and more numerous nuclear foci in myotubes. The RNA signal colocalized with PSPC1, and biochemical experiments linked the transcripts to NONO and other DBHS-family paraspeckle proteins. These observations provide direct muscle evidence for differentiation-dependent spatial association and molecular participation, corresponding to E2 for the C2C12 paraspeckle claim.

The same paper also provided strong architectural evidence, but the model boundary is important. Antisense-mediated depletion disrupted paraspeckles in most depleted cells, and transcriptional reactivation experiments placed the RNA early in paraspeckle reassembly. Those perturbation experiments were performed in HeLa cells, not in C2C12 myoblasts or myotubes. They therefore support the general architectural function of NEAT1, not direct proof that paraspeckle assembly is necessary for myogenic differentiation. A later study in human embryonic stem-cell differentiation similarly linked NEAT1 expression, paraspeckle formation, and nuclear retention of selected inverted-repeat-containing transcripts, but again outside skeletal muscle [38].

Domain-resolved work strengthened the non-muscle architectural model. NEAT1_2 contains separable regions that recruit NONO and SFPQ, promote assembly, and generate a core–shell organization; perturbations of these regions and in vitro assays support phase separation [10]. Block-copolymer and triblock-micelle models further explain how an RNA scaffold with distinct interaction domains can impose paraspeckle geometry [11,12]. These studies justify condensate language for paraspeckles as a general structure. They do not establish that a change in condensate material state mediates a specific muscle phenotype.

### 3.2. NEAT1-Dependent Myogenic Regulation Without Demonstrated Paraspeckle Mediation

Neat1 perturbation in C2C12 cells and mouse muscle provides direct evidence for myogenic function [19]. Knockdown reduced proliferation and promoted differentiation and fusion, whereas gain-of-function produced the opposite cellular tendency. In vivo depletion increased myofiber cross-sectional area but delayed cardiotoxin-induced regeneration, consistent with reduced expansion of PAX7-positive progenitors before differentiation. Mechanistically, Neat1 interacted with EZH2, and perturbation altered EZH2 or H3K27me3 occupancy at Cdkn1a, Myog, Myh4, and Tnni2. A mapped RNA segment and epistasis with EZH2 or P21 strengthen the chromatin-regulatory claim to E4, while the regeneration result reaches E5 for a Neat1-dependent muscle phenotype.

The study did not demonstrate that paraspeckle assembly mediated these effects. It did not quantify NEAT1_2 separately, measure paraspeckle number or dynamics after perturbation, or rescue the phenotype with isoform-specific constructs. The mapped 1001–1540 nucleotide region lies within sequence shared by the shorter and longer transcripts. Consequently, the supported conclusion is that Neat1 can regulate myogenic proliferation and differentiation through an EZH2-linked mechanism. Calling this a paraspeckle mechanism would silently add an untested causal step.

The apparently opposing cellular and tissue outcomes can coexist because they measure different stages. Earlier cell-cycle exit may increase short-term differentiation yet reduce the progenitor expansion required for timely regeneration. Fiber area, progenitor number, regeneration kinetics, and mature force are not interchangeable endpoints.

Cellular composition and myonuclear specialization add a second boundary. Single-nucleus RNA sequencing of mouse skeletal muscle across postnatal development and aging [39], and of nuclei isolated from uninjured, regenerating, and dystrophic myofibers [40], identified transcriptionally distinct myonuclear populations, including neuromuscular- and myotendinous-junction states. These data derive from muscle tissue rather than cultured myotubes. Whole-muscle measurements can therefore reflect changes in cell composition or nuclear subtype. Isoform-resolved in situ hybridization with cell-type markers and quantitative three-dimensional imaging is required to assign NEAT1 changes to a defined lineage and nuclear position.

Injury also changes inflammation, oxygenation, mechanical load, and progenitor state, any of which may induce NEAT1_2. A causal test should therefore measure the timing of paraspeckle assembly and RNA processing relative to these responses and compare rescue by assembly-competent and assembly-deficient NEAT1_2. This design would distinguish an adaptive stress response from an architectural requirement for regeneration.

### 3.3. PABPN1, MATR3, and Paraspeckle-Linked RNA Processing in Muscle Disease

Muscle disease studies provide a more direct link between NEAT1-centered processing and a defined perturbation. In primary mouse myoblasts, depletion of poly(A)-binding protein nuclear 1 (PABPN1) or MATR3 increased Neat1 stability and nuclear foci, redistributed SFPQ, and altered A-to-I editing of the paraspeckle-retained Ctn transcript [20]. Co-depletion of Neat1 shifted the editing phenotype toward control, placing Neat1 functionally downstream. The same study identified altered SFPQ distribution in an oculopharyngeal muscular dystrophy mouse model and nuclear–cytoplasmic redistribution of selected transcripts in PABPN1-deficient human myoblasts.

This evidence reaches beyond colocalization because it combines muscle models, perturbation, an RNA-processing readout, and genetic interaction. It does not determine whether the relevant variable is transcript abundance, NEAT1 isoform, protein recruitment, or paraspeckle material state. Matched molecule counting and isoform-specific rescue are required to separate these alternatives.

Taken together, Table 1 assigns E2 to differentiation-linked paraspeckle association in C2C12 cells, E4–E5 to NEAT1-dependent myogenic regulation, and E4–E5 to the PABPN1/MATR3 processing module. Table 2 places paraspeckle material evidence mainly in non-muscle systems. No direct muscle study yet shows that NEAT1_2-dependent assembly is necessary for a defined myogenic or disease phenotype.

## 4. MALAT1, Nuclear Speckles, and Context-Dependent RNA Processing

MALAT1 is a broadly expressed, abundant nuclear RNA. In muscle, it is best treated as a speckle-associated regulator whose effect depends on cell state and perturbation history, not as an obligatory scaffold or a uniformly pro- or anti-myogenic RNA.

### 4.1. A Regulatory Role at Speckles

In human cultured cells, MALAT1 associates with serine/arginine-rich splicing factors, changes their distribution between nucleoplasm and speckles, alters phosphorylation-associated pools, and affects a subset of alternative-splicing events [5]. These experiments provide a mechanistic route from a nuclear-retained lncRNA to splice-site choice. The route is not equivalent to structural maintenance of the speckle. Mouse knockout studies found preserved speckle morphology and generally normal development, although local transcriptional effects and context-specific phenotypes remained possible [6,17,18].

Recent biophysical studies refine this distinction. Speckle proteins can form compositionally distinct microphases, and MALAT1 can preferentially associate with particular serine/arginine-rich protein phases [34]. Under hypoxia in cancer cells, MALAT1 was reported to promote SRSF1 condensation near speckles and to alter pre-mRNA processing [41]. These findings make assembly state a plausible regulatory variable. They are non-muscle results obtained under specific conditions and should guide tests in myoblasts and myotubes.

### 4.2. Opposing Myogenesis Phenotypes

Two studies link MALAT1 to myogenesis under their tested schedules. In C2C12 and primary human myoblasts, MALAT1 increased during differentiation, whereas myostatin lowered its abundance. Acute silencing reduced proliferation through cell-cycle arrest [21]. A separate C2C12 study found impaired differentiation after MALAT1 silencing and proposed a miR-133–SRF competing-endogenous-RNA pathway [22]. The studies establish different perturbation-dependent outcomes and should not be treated as equivalent demonstrations of a direct differentiation mechanism. Neither establishes speckle-mediated myogenesis.

A third study produced the opposite directional result under an early-differentiation and constitutive-loss framework [23]. Malat1 declined soon after differentiation induction, and two loss-of-function approaches increased myogenic markers, Myogenin- or myosin-heavy-chain-positive cells, and MyoD-responsive reporters. Malat1 also recruited Suv39h1, HP1β, and HDAC1 to MyoD target genes. Constitutive deletion left baseline muscle largely intact but enhanced regeneration after cardiotoxin injury and in mdx mice. These findings support an inhibitory chromatin mechanism during an early differentiation window and provide E5 evidence for a conditional regeneration phenotype.

The contradiction cannot be resolved by averaging the studies. The protocols differed in cell density, sampling time, perturbation duration, knockdown magnitude, proliferation state, and endpoint. None of these variables was isolated across studies. They remain candidate moderators that require a matched comparison.

The proposed miR-133 competing-endogenous-RNA mechanism requires quantitative confirmation [22]. Reporter rescue and reciprocal expression do not establish whether endogenous MALAT1 binding-site abundance and occupancy are sufficient to alter miR-133 activity. Endogenous site mutation, absolute molecule counting, and rescue within the physiological expression range would test this model. The miR-181a pathway is mechanistically different. In that study, miR-181a and Ago2 promoted nuclear Malat1 degradation, which relieved repression of MyoD targets [23]. It should not be interpreted as evidence that Malat1 sequesters miR-181a.

The constitutive knockout raises a separate question. A normal baseline phenotype may indicate dispensability, developmental compensation, or insensitive endpoints. An inducible adult knockout paired with acute RNA degradation in satellite cells would test whether enhanced regeneration persists without developmental adaptation.

A decisive comparison would use the same myoblast preparation at matched density and measure MALAT1 through cell-cycle exit, commitment, and fusion. Rapid degradation, CRISPR interference, and constitutive loss should be paired with calibrated rescue and common endpoints for chromatin, SR-protein state, splicing, and myogenesis. A timing-dependent reversal of phenotype would support the temporal-state model.

### 4.3. The Unclosed Link Between MALAT1 and Muscle Splicing

The strongest MALAT1–splicing mechanism was established outside muscle, whereas the direct muscle studies emphasized proliferation, miRNA-linked regulation, chromatin occupancy, and differentiation. Among the primary muscle studies evaluated here, none combined MALAT1 perturbation with transcriptome-wide alternative-splicing analysis and quantitative speckle or SR-protein measurements. This is a bounded statement about the source set searched through 20 October 2026, not proof that no relevant study exists.

That gap matters because changes in myogenic markers can arise through several routes. MALAT1 may influence chromatin at MyoD-responsive genes, local concentrations of splicing factors, transcription elongation, RNA stability, or cell-cycle composition. A speckle-mediated mechanism should predict that an assembly-selective perturbation changes a defined splicing program even when total MALAT1 abundance and proliferation are controlled. A chromatin-mediated mechanism should instead track with local histone marks and promoter occupancy. Both mechanisms could operate at different stages.

Table 1 therefore separates E3–E5 muscle phenotypes from foundational non-muscle speckle evidence, while Table 2 records that gross speckles persist after MALAT1 loss. The direction of the myogenic phenotype remains context dependent, and no direct causal chain from MALAT1 through speckle state to muscle alternative splicing has been demonstrated in the searched source set.

## 5. Other Nuclear Muscle ncRNAs That Meet a Mechanistic Threshold

Additional muscle lncRNAs should enter this framework only when spatial localization is connected to a defined nuclear process; most chromatin-associated myogenic lncRNAs are regulatory candidates, not established nuclear-body scaffolds.

### 5.1. FRG2A/B Transcripts as a Nucleolar Disease Module

Nucleolar FRG2 transcripts provide the strongest additional compartment-centered module. In primary human myoblasts from individuals with facioscapulohumeral muscular dystrophy (FSHD), FRG2A/B transcripts were elevated and localized to a defined nucleolar region [24]. Chromatin isolation by RNA purification enriched rDNA and centromeric sequences, and FSHD cells showed increased associations between centromeres and nucleoli. These measurements support spatial participation in nucleolar and perinucleolar organization, while the high sequence identity of FRG2A and FRG2B limits paralog-specific assignment.

The functional chain extends beyond localization. FSHD myoblasts had reduced 45S pre-rRNA abundance, lower nascent nucleolar RNA synthesis, altered nucleolar number and area, and reduced nascent protein synthesis. Depletion of FRG2A/B transcripts increased 45S pre-rRNA and nascent RNA synthesis and reversed the protein-synthesis defect. Proteomics identified incomplete compensation during myotube differentiation, with persistent underrepresentation of several muscle-related proteins. These results support E4 evidence for a nucleolar rDNA-transcription mechanism in patient-derived cells.

The study does not establish that FRG2 transcripts create a separate condensate or that this mechanism explains the full clinical phenotype of FSHD. rDNA transcription, nucleolar morphology, translation, and disease severity were not all linked by an in vivo rescue. The supported model is narrower: D4Z4-associated derepression increases nucleolar FRG2 RNA, which contributes to impaired rRNA synthesis and translational capacity in the tested FSHD cells.

Assay interpretation further narrows the FRG2 model. Reduced 45S pre-rRNA and nucleolar 5-ethynyl uridine incorporation support impaired nascent rRNA production, whereas unchanged processing intermediates argue against a primary maturation defect in the assays used. Lower homopropargylglycine incorporation indicates reduced nascent protein synthesis, not enhanced protein degradation. Ribosome profiling and pulse–chase proteomics would be required to distinguish altered translation from protein stability or differentiation effects.

### 5.2. Chromatin-Associated lncRNAs as Boundary Cases

Ppp1r1b-lncRNA meets the mechanistic threshold through a chromatin-centered ribonucleoprotein complex rather than a named nuclear body. In mouse and human muscle models, the RNA interacted with polycomb repressive complex 2 (PRC2) and promoted myogenic gene expression; genetic studies linked it to cardiac and skeletal muscle development [25]. More recent work showed that hnRNPA1 stabilizes a Ppp1r1b-lncRNA–PRC2 complex that helps maintain muscle-progenitor identity [26]. These data support a nuclear chromatin-regulatory mechanism but not condensate terminology.

Three other lncRNAs meet the inclusion threshold through defined nuclear interactions rather than compartment assembly. Myoparr acts near the Myog locus [27]; Linc-RAM interacts with MyoD and supports a transcriptional complex at myogenic genes [28]; and Dum recruits DNA methyltransferases to repress Dppa2 during differentiation and regeneration [29]. MYH1G-AS provides an avian chromatin-associated example, but its species context and the absence of a named nuclear-body mechanism limit extrapolation to mammalian muscle [42]. These studies broaden the range of nuclear regulatory mechanisms without establishing new condensates.

Table 1 groups these RNAs as chromatin or nucleolar regulators and assigns evidence tiers according to perturbation and rescue. Table 2 includes only FRG2A/B because it is linked to a defined nucleolar domain. None of the boundary-case lncRNAs has the domain-resolved assembly or material-property evidence needed to define a new muscle nuclear condensate.

**Figure 2:**
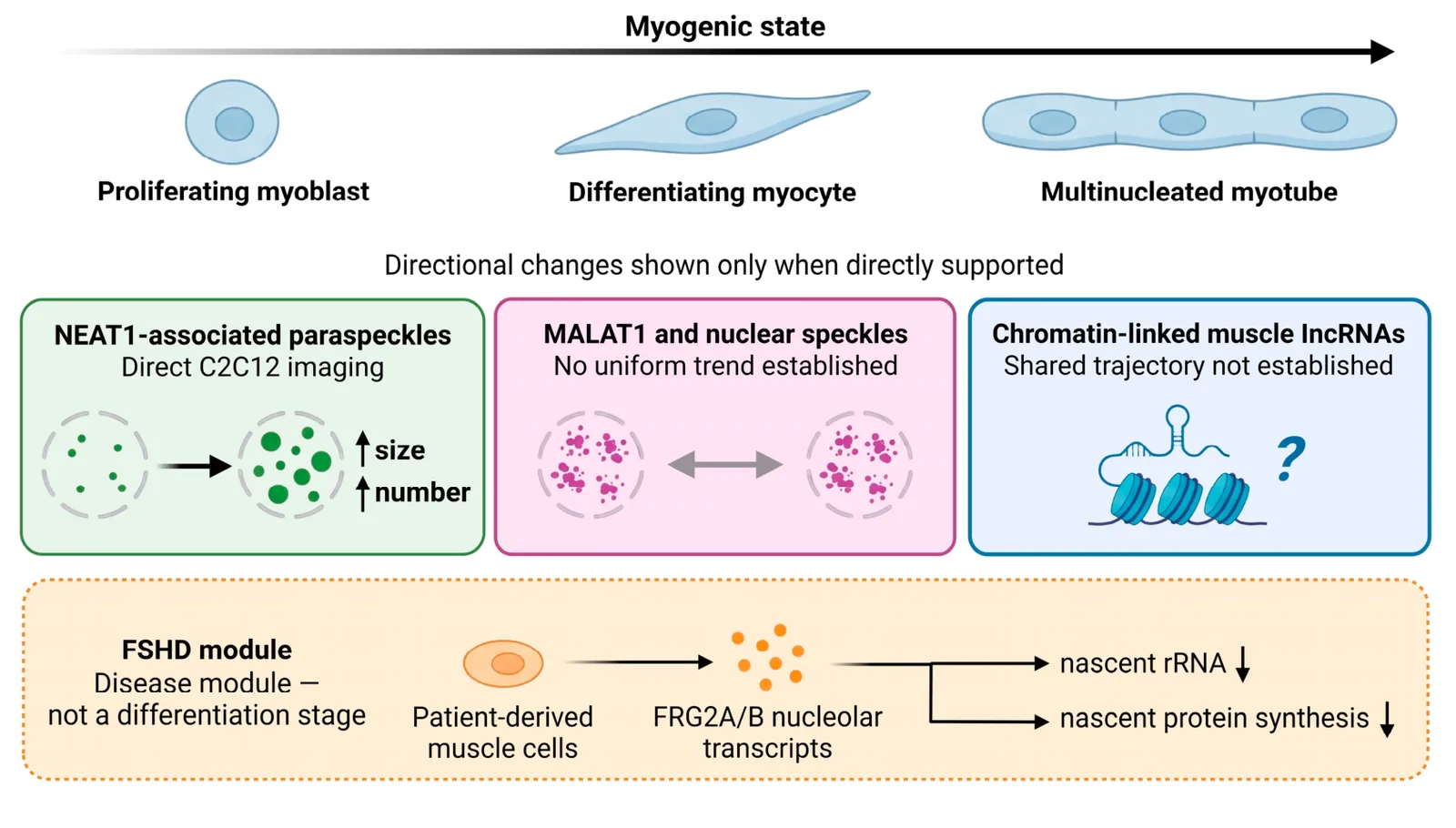
Evidence-supported nuclear RNA changes across myogenic states and a separate FSHD module. The upper sequence shows a proliferating myoblast, a differentiating myocyte, and a multinucleated myotube. In C2C12 cells, NEAT1-associated paraspeckle foci became larger and more numerous during differentiation [4]; this observation does not show that assembly causes myogenesis. MALAT1 studies report context-dependent and opposing myogenic phenotypes, so no uniform differentiation-associated direction is assigned [21,22,23]. Neat1–EZH2 and MYH1G-AS provide chromatin-linked examples [19,42]. Other promoter- or chromatin-associated regulators are summarized in Table 1. These RNAs have not been shown to follow one common stage-dependent trajectory. The lower panel is not part of the developmental sequence. It summarizes the FRG2A/B nucleolar module in FSHD patient-derived muscle cells, where FRG2A/B depletion corrected reduced nascent rRNA production and nascent protein synthesis; in vivo rescue has not been tested [24]. The top arrow indicates myogenic progression. Green, magenta, blue, and orange identify the NEAT1, MALAT1, chromatin-linked, and FSHD modules, respectively. The gray double-headed arrow indicates the absence of a uniform MALAT1 trend; the question mark indicates an unresolved shared trajectory for chromatin-linked RNAs. Upward and downward arrows indicate reported increases and decreases. Arrows in the FSHD module connect the cellular context, transcripts, and measured outcomes. Symbols denote reported localization or association and do not assign a material state. Created in BioRender. Lee, W. (2026) https://BioRender.com/cicyvwz.

Figure 2 integrates these findings across the myoblast-to-myotube sequence while keeping the FSHD nucleolar module separate from normal myogenic progression.

## 6. Pathological Repeat-RNA Assemblies in DM1 and DM2

Expanded CUG and CCUG RNAs cause disease through abnormal nuclear retention and loss of RNA-binding-protein function, but the visible structures range from single retained transcripts to multimeric assemblies; “RNA focus” is therefore the correct default term.

### 6.1. Genetic Lesions and Sequestration of Processing Factors

Myotonic dystrophy type 1 (DM1) is caused by a CTG expansion in the 3′ untranslated region of DMPK. The mutant transcript contains expanded CUG repeats. Early work showed repeat-length-dependent binding of muscleblind proteins and their accumulation with expanded RNA in nuclear foci from affected cells [7]. DM2 is caused by a CCTG expansion in intron 1 of CNBP (formerly ZNF9), producing expanded CCUG RNA and establishing a parallel toxic-RNA mechanism [32]. In both disorders, the mutation changes RNA behavior without requiring loss of the encoded protein to explain the core spliceopathy.

The most reproducible molecular consequence is reduced functional availability of muscleblind-like (MBNL) proteins. MBNL and CELF families normally regulate developmentally programmed RNA processing, often in opposing directions [43,44]. Expanded repeats recruit MBNL, while CELF1 activity can increase through separate signaling and post-translational mechanisms. Increased nuclear CELF1 activity is sufficient to produce muscle wasting in mice, demonstrating that redistribution or activation of a processing factor can drive tissue pathology without being a simple readout of an RNA focus [45].

Patient and model studies place the expanded RNAs near processing domains but do not make them physiological speckles. DM1 RNA accumulated at the periphery of nuclear speckles in cultured cells [46]. DM2 patient myoblasts and tissues contain CCUG RNA/MBNL1-positive foci [8,33]. These observations support spatial proximity to RNA-processing machinery and sequestration. They do not show that the foci share the composition, topology, or reversibility of a normal nuclear speckle.

### 6.2. From Phase Transition to Single Retained RNA

Purified repeat RNAs can undergo length- and concentration-dependent sol–gel transitions, and repeat-RNA foci in transfected cells were sensitive to interventions that disrupted intermolecular base pairing [35]. This work established biophysical plausibility for RNA-driven phase transitions and identified repeat length as a determinant of assembly. It is appropriate evidence for condensate-like behavior under the tested in vitro and cellular conditions.

Quantitative imaging in patient-derived DM1 myotubes produced a critical refinement. Most apparent foci corresponded to individual nuclear-retained expanded transcripts, while larger multimeric foci were less common, arose from transcriptional bursts, and dissipated over time [30]. Individual transcripts were sufficient to bind MBNL1, whereas multimeric focus formation depended on MBNL. Thus, sequestration and toxicity need not require a bulk RNA condensate. An image-limited punctum can represent one unusually large RNA–protein complex.

The two results are compatible only at a defined level. Repeat RNA has an intrinsic capacity for intermolecular assembly, but endogenous molecule number, transcriptional bursting, protein occupancy, and nuclear clearance determine whether that capacity is realized in a patient cell. The material state of one experimental construct cannot be assigned to every endogenous focus. Future studies should combine calibrated molecule counting, single-particle tracking, fluorescence recovery, perturbation of repeat–repeat pairing, and measures of MBNL availability in the same patient-derived muscle model.

This distinction changes how focus burden should be interpreted. Counting puncta can underestimate dispersed individual expanded transcripts, while integrated fluorescence can overestimate molecule number when probe accessibility varies with repeat length or protein occupancy. A treatment that converts a multimeric focus into several individual RNAs may reduce focus size without restoring MBNL availability. Conversely, degradation of individual transcripts can improve splicing without a dramatic change in the number of large foci if a transcription site remains active. Molecular imaging should therefore be calibrated against transcript abundance and paired with a functional sequestration readout.

### 6.3. Spliceopathy, Developmental State, and Tissue Specificity

Transcriptome studies of DM skeletal muscle and heart show extensive but tissue-dependent changes in alternative splicing and gene expression [47]. Congenital DM1 disrupts prenatal RNA processing and myogenesis, placing the mechanism within a developmental window, not only in degenerating adult muscle [48]. DM1 and DM2 are not molecular duplicates: competition between rbFOX1 and MBNL1 for CCUG RNA and comparisons of human cellular models identify subtype-specific processing signatures [36,49].

Specific splicing events connect loss of processing-factor activity to physiological output. Mis-splicing of CACNA1S changes CaV1.1 channel gating and contributes to weakness [50]. Combined loss of MBNL1 and MBNL2 in mice produces severe cardiac electrical and processing phenotypes, while zebrafish mbnl mutants reproduce molecular and physical features across tissues [51,52]. These models support MBNL loss of function as a causal layer, although no single model reproduces the repeat mutation, multisystem distribution, and temporal progression of human disease.

Human studies further show that molecular abnormalities vary with developmental and functional context. In children with myotonic dystrophy, composite splicing changes were associated with physical performance [53]. Hybrid myotubes containing patient and unaffected nuclei modified several cellular hallmarks, showing that a shared cytoplasm can alter phenotype without correcting every nucleus [54]. Differentiation itself intensified repeat-RNA phenotypes in DM1 models [55]. These observations support cell state and nuclear context as mechanistic variables rather than technical nuisances.

Splicing signatures also require causal discipline. An exon that correlates with disease severity can be a sensitive biomarker without mediating weakness. Conversely, a physiologically important event may be difficult to measure reproducibly in a small biopsy. Mechanistic prioritization should combine direct MBNL or CELF binding, perturbation of the candidate exon, protein-level confirmation, and a relevant physiological readout. This standard is met most closely for selected ion-channel events; it remains incomplete for many transcriptome-wide changes.

Regeneration is another causal layer. Experimental reduction of MBNL activity impairs aspects of skeletal muscle regeneration, while a repeat-RNA mouse model showed loss of PAX7-positive satellite cells, delayed maturation, fibrosis, and partial correction by a DMPK-targeting oligonucleotide [56,57]. Congenital DM models also show altered myokine signaling [58]. These studies extend spliceopathy beyond contraction toward progenitor maintenance and repair, but they do not imply that every regenerative defect results from the same exon-level event.

Spatial distribution varies within muscle. Ribonuclear foci occur at neuromuscular junctions, and sense and antisense DMPK foci show developmental and tissue-specific patterns [59,60]. Abnormal nuclear aggregation has been linked to myotube degeneration in DM1 models [61]. In DM2, multi-level profiling points to mitochondrial dysfunction [62]. Separate CCUG-repeat studies implicate chloride-channel splicing [63], reduced global protein synthesis [64], repeat-associated non-AUG translation [65], and apoptosis [66]. These findings support a shared toxic-RNA core with disease- and tissue-specific downstream branches.

DM2 requires particular restraint because its average expansion can be much longer than the DM1 repeat, yet clinical anticipation and tissue patterns differ. The CCUG repeat is excised from an intron, and the fate of the host transcript, excised repeat RNA, and associated proteins need not match that of the DMPK 3′ untranslated region. Evidence for MBNL sequestration is strong, but contributions from CNBP dosage, RAN translation, and other RNA-binding proteins remain incompletely separated. A therapy effective against CUG RNA cannot be assumed to bind, traffic, or degrade CCUG RNA with the same selectivity.

Human genetics, patient tissue, and multiple animal models establish repeat-RNA toxicity and MBNL-dependent spliceopathy. The number, composition, and material state of individual RNA foci remain variable. Condensate terminology is justified for experiments that directly measure phase or gel behavior, not as a default description of all DM1 or DM2 puncta.

**Figure 3:**
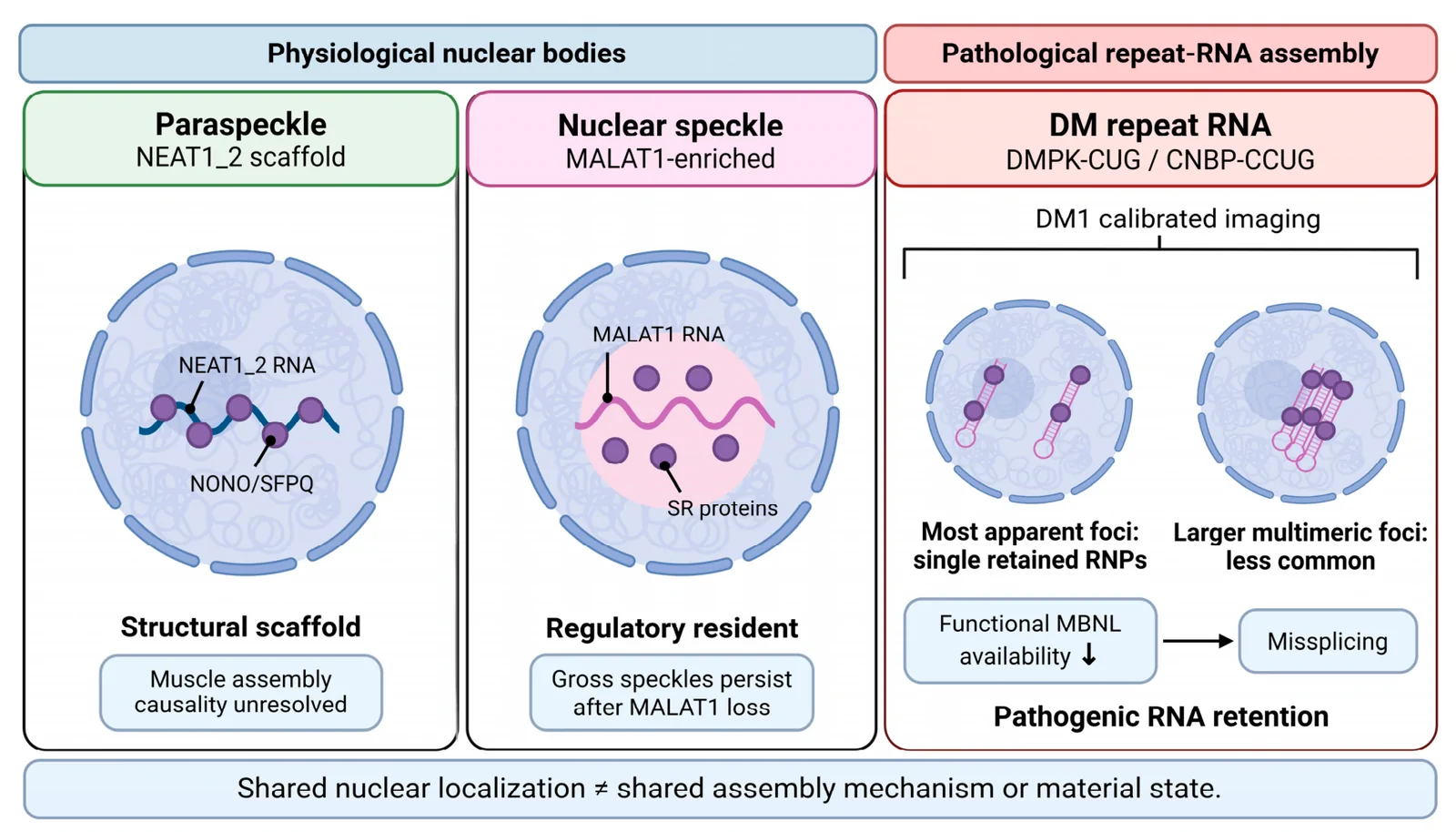
Physiological nuclear compartments and pathological repeat-RNA assemblies. The left panel shows NEAT1_2 as the architectural paraspeckle scaffold associated with NONO and SFPQ; direct assembly-to-phenotype causality remains unresolved in skeletal muscle. The center panel depicts MALAT1 enrichment with serine/arginine-rich (SR) proteins in nuclear speckles. MALAT1 may influence regulatory partitioning, but gross speckles persist after MALAT1 loss. The right panel shows retained DMPK transcripts containing expanded CUG repeats and excised CNBP intronic RNAs containing expanded CCUG repeats. These RNAs bind MBNL, reduce its functional availability, and contribute to spliceopathy. In the examined patient-derived DM1 myotube model, calibrated imaging indicated that most apparent foci were individual retained ribonucleoprotein particles, whereas larger multimeric foci were less frequent. The bracketed single-particle comparison is specific to DM1 and is not assigned to DM2. The systems share nuclear localization but not an established assembly mechanism or material state; the drawing is schematic. Created in BioRender. Lee, W. (2026) https://BioRender.com/dzqaypf.

Figure 3 compares these spatial systems side by side so that architectural, regulatory, and pathogenic roles are not conflated.

## 7. Matching Interventions to Causal Layers

Therapeutic evidence is strongest when an intervention reaches the intended molecular target, corrects an RNA-processing defect, and improves a relevant muscle phenotype. A change in focus number or RNA abundance alone is not sufficient.

### 7.1. Toxic-RNA Reduction and Clinical Translation

Systemic antisense oligonucleotides (ASOs) targeting nuclear-retained CUG-expanded RNA reduced toxic RNA in the HSA^LR^ mouse model and corrected splicing, myotonia, histopathology, and transcriptomic abnormalities [31]. This experiment remains a strong preclinical benchmark because target engagement, molecular rescue, and tissue phenotypes were measured in the same study.

Human studies have made muscle delivery the central translational constraint. Baliforsen reached skeletal muscle in a phase 1/2a trial, but exposure was below that predicted to produce substantial DMPK reduction [67]. A transferrin-receptor-1-targeted antibody–siRNA conjugate subsequently reduced DMPK RNA and improved a composite missplicing score in some phase 1/2 dose groups [68,69]. However, the sponsor-reported phase III HARBOR analysis did not show a significant benefit for the primary functional endpoint [70]. These results establish human target engagement and molecular activity, but not clinical efficacy.

Splicing is useful as a pharmacodynamic bridge when several validated MBNL-responsive exons are measured together. In mouse models, a targeted panel showed graded responses to repeat RNA, MBNL dosage, and ASO treatment [71]. Such biomarkers can compare upstream interventions, but they do not replace controlled functional outcomes.

### 7.2. Interventions at Other Causal Layers

Programmable nucleases can act on repeat RNA, repeat transcription, or the DNA expansion itself. RNA-targeting Cas9 removed repeat RNA and corrected protein localization and splicing in cellular models [72]. Transcriptional blockade with catalytically inactive Cas9 and genome-editing approaches improved molecular or physiological outcomes in patient cells and mice [73,74,75]. Their main unresolved requirements are distributed-muscle delivery, allele selectivity, off-target control, immune compatibility, and durability.

Repeat structure also provides a pharmacological target. Structure-informed degraders and RNA-cleaving dimers reduced expanded CUG RNA or corrected processing defects in patient-derived myotubes and mouse models [76,77,78]. Erythromycin and furamidine altered repeat occupancy or MBNL-related outcomes, although their effects were not restricted to a single molecular mechanism [79,80]. Exposure and transcriptome-wide specificity remain separate translational tests.

Other representative strategies act on repeat binding or processing capacity. An engineered pentatricopeptide-repeat protein improved RNA toxicity in a mouse model [81]. Splice-switching oligonucleotides and AAV-delivered U7 small nuclear RNAs target RNA processing or abundance [82,83]. AntimiR-mediated and direct MBNL restoration improved processing phenotypes in experimental systems [84,85]. These approaches are summarized by causal target in Table 3 rather than catalogued individually in the text.

Table 3 identifies the cellular or molecular target first, then records the representative strategy, evidence stage, demonstrated result, and principal limitation.

**Figure 4:**
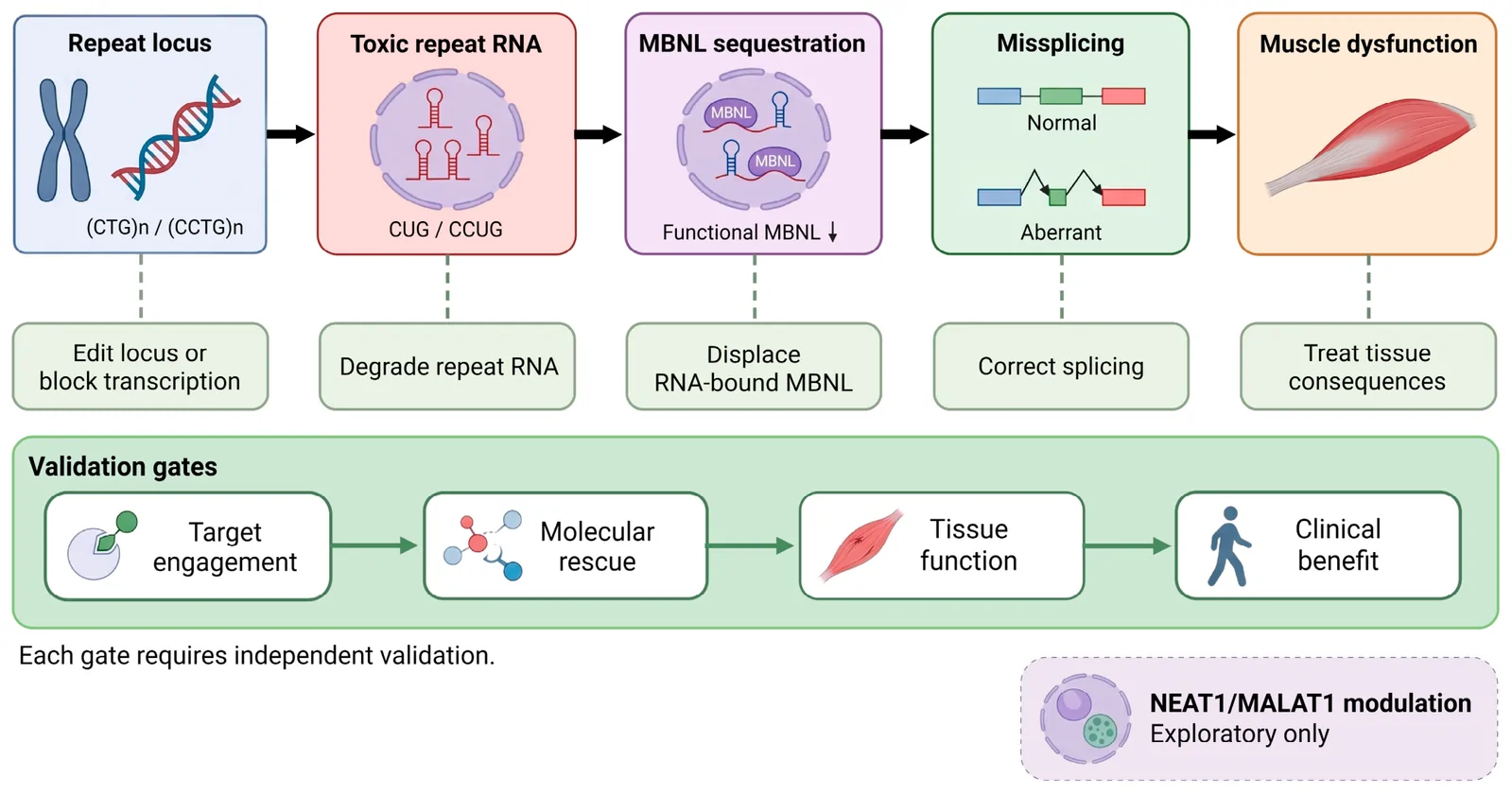
Intervention points and independent validation gates for repeat-RNA disease. The upper pathway summarizes the shared toxic-RNA core of myotonic dystrophy: expanded CTG or CCTG DNA, expanded CUG- or CCUG-repeat RNA, MBNL sequestration, missplicing, and muscle dysfunction. This sequence does not exclude parallel disease- or tissue-specific mechanisms. Dashed green connectors locate candidate interventions and do not indicate activation or established efficacy. The interventions include editing the repeat locus or blocking its transcription, degrading repeat RNA with ASOs, siRNAs, or RNA-cleaving approaches, displacing MBNL from repeat RNA or restoring MBNL, correcting splicing, and treating tissue consequences. The lower row separates target engagement, molecular rescue, tissue function, and clinical benefit; each gate requires independent validation. The separate dashed module identifies NEAT1/MALAT1 modulation as exploratory. Such modulation would require evidence of the intended processing correction and a favorable muscle phenotype without disruption of normal nuclear function. Solid black arrows depict the conceptual pathogenic sequence. Solid green arrows order the validation gates without implying that success at one gate establishes the next. Background colors distinguish pathway stages or functional groups and do not encode evidence strength. Colored rectangles in the splicing diagrams represent exons, and angled connectors represent alternative splice choices. The downward arrow denotes reduced functional MBNL availability. ASO, antisense oligonucleotide; MBNL, muscleblind-like protein; siRNA, small interfering RNA. Created in BioRender. Lee, W. (2026) https://BioRender.com/ic17bow.

Therapeutic claims should remain at the level directly demonstrated. Target engagement can be measured as repeat-RNA reduction, repeat occupancy, MBNL release, or editing at the intended locus. Molecular rescue requires restored functional MBNL or a validated processing signature; tissue and clinical rescue require relevant muscle and controlled patient outcomes, respectively. Modulation of physiological NEAT1- or MALAT1-associated compartments remains exploratory and would additionally require preservation of normal nuclear function. Figure 4 maps representative interventions to successive causal layers and separates these independent validation gates.

## 8. Unresolved Questions and Experimental Standards

### 8.1. Evidence Required for an Architectural RNA Role in Muscle

Assigning an architectural role requires separation of RNA abundance from assembly state. For NEAT1, an informative experiment would deplete endogenous Neat1, rescue NEAT1_1 or NEAT1_2 at matched abundance, and compare wild-type NEAT1_2 with a domain mutant that retains stability but cannot organize paraspeckles. Quantitative imaging would measure molecule number, paraspeckle number, size, and exchange. RNA sequencing and retention assays would define processing outputs, while differentiation, regeneration, and force would provide muscle endpoints. A phenotype rescued only by assembly-competent NEAT1_2 would support architectural causality.

For MALAT1, the decisive design must be temporal. Rapid degradation and reversible rescue would minimize developmental compensation. Density-matched myoblasts would be sampled before cell-cycle exit, during commitment, and during fusion. Speckle morphology, SR-protein microphase partitioning, phosphorylation, nascent transcription, chromatin occupancy, and splicing would be measured in the same cells or matched aliquots. If a processing phenotype follows altered protein partitioning while chromatin occupancy remains stable, the speckle route gains support; the converse result favors a chromatin mechanism.

For repeat RNA, counting endogenous molecules is essential. RNA FISH protocols and expanded-repeat reverse transcription require calibration because probe accessibility and reverse-transcriptase processivity can bias the inferred abundance [86,87,88]. Live-cell imaging should distinguish individual RNAs, transcription-site bursts, and multimeric foci. Material-property perturbations must be separated from global RNA depletion or stress. The most informative endpoint is functional MBNL availability linked to a processing signature and muscle phenotype.

Model selection should follow the claim. C2C12 cells are useful for controlled differentiation and imaging but do not reproduce the multinucleated architecture, loading, innervation, or aging of adult human muscle. Primary patient myoblasts preserve disease alleles but vary in repeat length, passage, epigenetic history, and differentiation capacity. Mouse models enable regeneration and force measurements but can differ in repeat context, transcript level, and tissue distribution. Human biopsy establishes clinical relevance yet offers limited perturbation and spatial sampling. Strong conclusions use these models as complementary tests without treating one as a universal standard.

Negative results should be equally explicit. Failure to detect a compartment change is informative only if the assay could resolve the expected magnitude, measured the relevant isoform, and included the proposed transition in the observation window. Failure to rescue can reflect incorrect mechanism, inadequate expression, or a rescue construct that lacks proper processing or localization. Predefined positive controls, blinded imaging, and reporting of detection limits are necessary before absence is interpreted as biological dispensability.

### 8.2. Falsifiable Tests of Nuclear RNA Mechanisms

Table 4 links each contested claim to a discriminating experiment and a result that would weaken the proposed mechanism.

These experiments are falsifiable. If NEAT1_2-specific assembly rescue does not restore the muscle phenotype, paraspeckle architecture is unlikely to be the required causal layer. If MALAT1 timing does not alter the sign of the differentiation phenotype under matched conditions, temporal state cannot explain the published contradiction. If endogenous DM1 RNAs remain predominantly single molecules across repeat lengths and transcription rates, a bulk condensation model would have limited relevance to sequestration. If molecular correction consistently fails to improve a mature muscle endpoint despite adequate exposure and duration, researchers must address downstream tissue damage or parallel mechanisms.

These standards also constrain extrapolation across repeat-expansion disorders. The broader literature identifies common principles involving repeat structure, RNA-binding-protein redistribution, and multiple downstream processing defects, while emphasizing the disease-specific effects of sequence, genomic locus, tissue, and developmental context [89,90]. Therapeutic reviews document a wide portfolio of modalities without resolving which combination will provide durable multisystem benefit [91]. DM1 and DM2 remain clinically and molecularly heterogeneous diseases and cannot be reduced to a single focus-formation disorder.

## 9. Conclusions

Nuclear noncoding RNAs influence skeletal muscle through several spatial mechanisms that should not be reduced to a single condensate model. NEAT1 links differentiation to paraspeckle association, chromatin regulation, and PABPN1/MATR3-dependent RNA processing, but isoform-specific architectural causality in muscle remains to be shown. MALAT1 regulates nuclear processes and muscle state without being required for gross speckle formation, and its opposing myogenesis phenotypes require a controlled temporal comparison. FRG2 transcripts connect a nucleolar RNA to rDNA transcription and translational capacity in FSHD cells. Expanded DMPK and CNBP transcripts cause toxicity by retaining RNA and reducing functional MBNL availability; calibrated imaging further shows that, in the examined patient-derived DM1 myotube model, most apparent foci correspond to individual retained transcripts rather than large multimeric assemblies.

The most useful unifying principle is therefore causal layering. Spatial localization identifies where a mechanism may occur. Perturbation and rescue determine whether an RNA or assembly is required. In vivo and patient evidence establish physiological relevance. Target engagement plus molecular and tissue rescue define translational progress. Applying these layers consistently will clarify which nuclear structures organize normal muscle RNA processing, which assemblies reflect pathology, and which intervention points are sufficiently supported for therapy.

## Figures and Tables

**Table 1:** Evidence-graded nuclear ncRNA modules in skeletal muscle.

RNA/Module	Nuclear Context	Model or Evidence Context	Perturbation and Measured Output	Tier	Interpretation Supported
NEAT1	Paraspeckle-associated	C2C12 myoblasts and myotubes	Differentiation-linked induction; RNA FISH; PSPC1/NONO association [4]	E2	Spatial and molecular participation in muscle
Neat1	Chromatin/EZH2	C2C12; mouse injury	Loss and gain of function; mapped EZH2 interaction; regeneration phenotype [19]	E4–E5	Neat1-dependent myogenesis; paraspeckle mediation untested
Neat1	PABPN1/MATR3-linked paraspeckle processing	Primary mouse and human myoblasts; OPMD model	PABPN1 or MATR3 depletion; SFPQ redistribution; editing and transport readouts [20]	E4–E5	Functional NEAT1-centered processing module
MALAT1	Nuclear speckle-associated	Non-muscle mechanism; mouse genetics	SR-factor partitioning and splicing [5]; preserved gross speckles in mouse loss-of-function studies [6,17,18]	E3; E5 *	Regulatory role; not an obligatory architectural RNA
Malat1	Chromatin and myogenic state	C2C12; mouse regeneration	Opposing acute phenotypes; constitutive loss enhances regeneration [21,22,23]	E3–E5	Context-dependent muscle regulator
FRG2A/B	Nucleolus/rDNA	FSHD patient myoblasts and myotubes	RNA localization, ChIRP, depletion, nascent rRNA and translation rescue [24]	E4	Nucleolar regulator of rDNA transcription in patient cells
Ppp 1b-lncRNA	Chromatin/PRC2 RNP	Mouse and human progenitors	Genetic perturbation; PRC2 and hnRNPA1 interaction [25,26]	E4–E5	Chromatin RNP; no named body established
Myoparr; Linc-RAM; Dum	Promoter- or chromatin-associated	Myoblasts; selected mouse models	RNA loss, partner mapping, and myogenic output [27,28,29]	E3–E5	Nuclear transcriptional regulators
DMPK CUG RNA	Single retained RNPs and RNA foci	Patient muscle cells; mouse models	MBNL recruitment, splicing, physiology, and therapeutic rescue [7,30,31]	E5–E6	Pathogenic RNA assembly; material state varies
CNBP CCUG RNA	RNA/MBNL foci	DM2 patient cells and tissue	Genetic lesion, MBNL sequestration, subtype-specific processing [8,32,33]	E5	Pathogenic RNA focus; not equivalent to DM1 in every downstream effect

Abbreviations: ChIRP, chromatin isolation by RNA purification; E, evidence tier; EZH2, enhancer of zeste homolog 2; FISH, fluorescence in situ hybridization; hnRNPA1, heterogeneous nuclear ribonucleoprotein A1; MATR3, matrin 3; ncRNA, noncoding RNA; NONO, non-POU domain-containing octamer-binding protein; OPMD, oculopharyngeal muscular dystrophy; PABPN1, poly(A)-binding protein nuclear 1; PRC2, polycomb repressive complex 2; PSPC1, paraspeckle component 1; RNP, ribonucleoprotein; SFPQ, splicing factor proline- and glutamine-rich. Evidence-tier entries apply to the claim stated in each row and do not represent a global score for a study. * For MALAT1, E3 applies to regulatory/splicing effects; E5 evidence limits an obligatory architectural role in vivo.

**Table 2:** Physiological nuclear compartments and pathological RNA assemblies.

System	Principal RNA Driver or Resident	Proteins	Assembly or Material Evidence	Processing Effect	Muscle-Specific Evidence Boundary
Paraspeckle	NEAT1_2 scaffold; NEAT1_1 partly outside	NONO, SFPQ, PSPC1, HNRNPK	NEAT1_2 processing and domain mapping [3,10,13]; micelle model [11]	Retention, editing, protein availability	Direct association and processing in muscle; material tests mainly non-muscle
Nuclear speckle	MALAT1-enriched resident, not obligatory scaffold	SR proteins, SON, SRRM2 and others	Multilayer architecture; protein microphases; context-dependent MALAT1 effects [16,34]	Splicing-factor state and transcript processing	Direct myogenic phenotypes; muscle speckle-to-splicing causality unresolved
Nucleolus in FSHD	FRG2A/B transcripts	rDNA/chromatin-associated factors	Spatial domain and chromatin association; no material-property test [24]	45S pre-rRNA synthesis and translational capacity	Direct patient-derived muscle-cell evidence; in vivo rescue absent
DM1 expanded RNA	DMPK CUG-expanded transcript	MBNL proteins; CELF dysregulation	In vitro sol–gel transition; endogenous population dominated by individual retained RNAs [30,35]	Alternative splicing, retention, transcriptional and regenerative effects	Human and in vivo causality strong; focus material state heterogeneous
DM2 expanded RNA	Excised CNBP CCUG-expanded RNA	MBNL, rbFOX1 and additional RBPs	Discrete RNA/MBNL foci; no universal material-property assignment [8,36]	Overlapping and subtype-specific spliceopathy	Human tissue evidence; fewer intervention studies than DM1

Abbreviations: CELF, CUGBP Elav-like family; DM1, myotonic dystrophy type 1; DM2, myotonic dystrophy type 2; MBNL, muscleblind-like; RBP, RNA-binding protein; rbFOX1, RNA-binding fox-1 homolog 1; rDNA, ribosomal DNA; SON, SON DNA- and RNA-binding protein; SR, serine/arginine-rich; SRRM2, serine/arginine repetitive matrix 2.

**Table 3:** Therapeutic strategies organized by causal target.

Causal Target	Representative Strategy	Evidence Stage	Demonstrated Result	Principal Limitation
Expanded CUG RNA	Systemic ASO	HSA^LR^ mouse [31]	RNA reduction with rescue of splicing, myotonia, histology, and transcriptome	Mouse study only [31]
DMPK RNA in human muscle	Baliforsen; TfR1-targeted siRNA	Phase 1/2 [67,68,69]; phase III update [70]	Muscle exposure and molecular activity; phase III primary endpoint not met	Clinical efficacy and multisystem exposure
Repeat transcription or DNA tract	dCas9 blockade or genome editing	Patient cells and mice [73,74,75]	Lower repeat-RNA burden with molecular or physiological rescue in models	Delivery, off-targets, and durability
Expanded repeat RNA	RNA-targeting Cas9	Patient myotubes and cellular models [72]	Repeat-RNA removal with protein relocalization and splicing correction	Vector delivery and immune constraints
Repeat RNA structure	Small-molecule cleaver or degrader	Patient myotubes and mice [76,77,78]	RNA or missplicing correction with selected functional rescue	Exposure and transcriptome-wide selectivity
CUG–MBNL occupancy	Small-molecule repeat binder	Cells and mice [79,80]	Protein redistribution, splicing correction, and myotonia improvement in models	Multimodal action and human exposure
Repeat RNA or processing	Engineered binder, splice switcher, or AAV–U7snRNA	Mouse model [81]; patient-derived or cellular models [82,83]	Reduced toxicity or correction of RNA-processing defects	Delivery, durability, and vector-specific risk
Functional MBNL	AntimiR or MBNL expression	Cells and mice [84,85]	Higher functional MBNL with processing rescue	Dose control and broad MBNL targets
Physiological compartments	NEAT1 or MALAT1 modulation	Exploratory	No mature therapeutic evidence	Pleiotropy and unresolved causal assembly feature

Abbreviations: AAV, adeno-associated virus; ASO, antisense oligonucleotide; dCas9, catalytically inactive Cas9; DMPK, dystrophia myotonica protein kinase; HSA^LR^, human skeletal actin long-repeat mouse model; MBNL, muscleblind-like; TfR1, transferrin receptor 1.

**Table 4:** Contested claims and decisive experiments.

Contested Claim	Evidence Supporting It	Evidence Limiting It	Likely Moderator	Decisive Experiment and Interpretation
NEAT1-dependent myogenesis is a paraspeckle mechanism	C2C12 paraspeckle association; NEAT1 perturbation changes myogenesis [4,19]	Assembly loss and muscle phenotype were not tested together; isoform unresolved	NEAT1_1 versus NEAT1_2; cell state; stress	Matched isoform and assembly-deficient rescue. Only assembly-competent NEAT1_2 rescue supports architectural causality.
MALAT1 uniformly promotes or inhibits differentiation	Each direction has direct perturbation evidence [21,22,23]	Protocols differ in timing, density, perturbation duration, and endpoint	Early versus late state; proliferation; adaptation	Time-resolved, density-matched acute and constitutive perturbations with physiological rescue.
MALAT1 is a speckle scaffold in muscle	Speckle enrichment and SR-factor regulation [5]	Speckles persist in knockout mice [6,17,18]	Stress- and protein-state-dependent microphases	Assembly-selective perturbation at fixed MALAT1 abundance paired with muscle splicing readouts.
Every DM RNA focus is a condensate	Repeat RNA undergoes phase transition in vitro and in transfected cells [35]	Most apparent foci in the examined patient-derived DM1 myotube model are individual retained RNAs [30]	Repeat length, transcriptional burst, protein occupancy	Endogenous molecule counting plus dynamics and material perturbation across repeat lengths.
FRG2 nucleolar RNA is a major driver of FSHD	Patient-cell depletion rescues rRNA and protein synthesis [24]	No in vivo rescue or direct clinical linkage; paralog resolution limited	D4Z4 context, differentiation, rDNA dosage	Paralog-specific perturbation and rescue in human muscle tissue or an appropriate in vivo model.
Loss of foci demonstrates therapeutic mechanism	Several treatments reduce foci with molecular rescue	Focus number can change independently of RNA number or MBNL availability	Probe access, RNA fragmentation, assembly state	Calibrated RNA counting, MBNL availability, splicing, and function in the same dose series.

Abbreviations: DM, myotonic dystrophy; MALAT1, metastasis-associated lung adenocarcinoma transcript 1; NEAT1, nuclear paraspeckle assembly transcript 1; RNA, ribonucleic acid.

## Data Availability

No new datasets were generated in this study. All information evaluated in this review is available in the cited publications, public clinical trial registries, and the cited sponsor report.

## Supplementary Materials

The following supporting information can be downloaded at: https://www.mdpi.com/article/10.3390/ijms27219713/s1.

## Abbreviations

The following abbreviations are used in this manuscript:

AAV	adeno-associated virus
ASO	antisense oligonucleotide
CELF	CUGBP Elav-like family
ChIRP	chromatin isolation by RNA purification
CNBP	CCHC-type zinc finger nucleic acid-binding protein
CRISPR	clustered regularly interspaced short palindromic repeats
DM1	myotonic dystrophy type 1
DM2	myotonic dystrophy type 2
DMPK	dystrophia myotonica protein kinase
FISH	fluorescence in situ hybridization
FSHD	facioscapulohumeral muscular dystrophy
HDAC	histone deacetylase
lncRNA	long noncoding RNA
MBNL	muscleblind-like
NEAT1	nuclear paraspeckle assembly transcript 1
NONO	non-POU domain-containing octamer-binding protein
PABPN1	poly(A)-binding protein nuclear 1
PRC2	polycomb repressive complex 2
PSPC1	paraspeckle component 1
RBP	RNA-binding protein
rDNA	ribosomal DNA
RNP	ribonucleoprotein
SFPQ	splicing factor proline- and glutamine-rich
SR	serine/arginine-rich
TfR1	transferrin receptor 1
