# Supplementary material for: Noncoding RNAs in Nuclear Condensates and RNA Processing in Skeletal Muscle
Source: Int J Mol Sci. 2026 Oct 29;27(21):9713. doi: 10.3390/ijms27219713 (PMC13763116; doi:10.3390/ijms27219713)
Supplement: Supplementary file 1 [file ijms-27-09713-s001.zip › ijms-4630310-supplementary.pdf]

Supplementary Methods S1

Literature identification, scope, and source verification

S1.1. Review design and scope

This article was prepared as a structured critical narrative review rather than a prospectively registered systematic review. The search and audit were designed to support a question-driven comparison of physiological nuclear RNA compartments and pathological repeat-RNA assemblies in skeletal muscle. The review therefore prioritized mechanistic studies that connected a defined RNA, nuclear location or assembly, perturbation, measured RNA-processing output, and muscle-relevant phenotype. It did not seek exhaustive coverage of all muscle noncoding RNAs or quantitative pooling of effect estimates.

Included domains were skeletal myogenesis, myofiber maturation, muscle regeneration, and neuromuscular disease; NEAT1-associated paraspeckles; MALAT1-associated nuclear speckles; spatially defined nucleolar or chromatin-linked muscle long noncoding RNAs; alternative splicing, nuclear retention, transcriptional organization, and RNA-binding-protein availability; DM1 and DM2 repeat-RNA foci; and interventions causally linked to these processes. Cytoplasmic competing-endogenous-RNA networks, extracellular-vesicle RNA, expression-only biomarker studies, and broad noncoding-RNA catalogues without a nuclear mechanism were excluded from the central synthesis.

S1.2. Information sources and search update

PubMed-indexed primary literature was identified through targeted combinations of the concept blocks in Table S1. Citation chaining from mechanistically central primary studies and recent focused reviews was used to identify foundational or older studies. PubMed and linked publisher records were used to verify bibliographic identity, final publication status, and correction or retraction notices. Official trial-registry or sponsor records were used only when a current trial-status result had not yet been reported in a peer-reviewed full article, and such evidence was labeled as sponsor-reported. The literature search cutoff was 20 October 2026. Selected source and publication-status records were rechecked during revision on 22 October 2026.

A representative PubMed query was: (("skeletal muscle" OR myoblast OR myotube OR myogenesis OR "muscle regeneration" OR myopathy OR "myotonic dystrophy") AND (NEAT1 OR MALAT1 OR FRG2 OR "noncoding RNA" OR "repeat RNA" OR DMPK OR CNBP OR CUG OR CCUG) AND (paraspeckle OR "nuclear speckle" OR nucleolus OR "RNA focus" OR condensate OR splicing OR "nuclear retention")). Terms were adapted for targeted updates on individual RNAs, diseases, assays, or interventions; the query was not treated as a single exhaustive search string.

Table S1. Search concept blocks used for targeted identification.

Concept block	Representative terms
Muscle	skeletal muscle; myoblast; myotube; myogenesis; muscle regeneration; myopathy; myotonic dystrophy
Nuclear organization	nuclear body; nuclear compartment; paraspeckle; nuclear speckle; nucleolus; RNA focus; condensate; phase separation
RNA	noncoding RNA; lncRNA; NEAT1; MALAT1; FRG2; repeat RNA; DMPK; CNBP; CUG; CCUG

Concept block	Representative terms
Processing and proteins	alternative splicing; nuclear retention; RNA processing; RNA-binding protein; MBNL; CELF; NONO; SFPQ; PSPC1
Intervention	antisense oligonucleotide; siRNA; RNA targeting; CRISPR; repeat transcription; small molecule; splice correction

S1.3. Eligibility and prioritization

Original studies using myoblasts, myotubes, isolated or intact skeletal muscle, animal muscle, or patient-derived muscle material were prioritized. Foundational non-muscle studies were retained when they established an architectural mechanism, domain requirement, molecular interaction, or material-property test that had not been performed in muscle. These studies were identified as non-muscle evidence and were not used to imply that the same mechanism operated in skeletal muscle without an independent muscle experiment.

Expression-only studies were not used to support architectural or processing claims. Other nuclear muscle noncoding RNAs entered a principal mechanistic module only when evidence reached at least physical interaction or component mapping and connected the RNA to a defined nuclear process. Therapeutic studies were prioritized when they demonstrated target engagement together with correction of a downstream RNA-processing defect or a relevant muscle phenotype. Studies reporting only biomarker association, focus number, or RNA abundance were interpreted at that measured level.

S1.4. Screening, extraction, and evidence assignment

Records were screened against the stated scope, and retained primary sources were reviewed for the species and model, cell or differentiation state, RNA identity and isoform when reported, spatial assay, perturbation, comparator, direct measured output, rescue or epistasis experiment, muscle phenotype, relevant negative result, and limitation on interpretation. Final peer-reviewed versions were used when available; a duplicate preprint was not retained when its final article was available.

Evidence tiers were assigned to individual claims rather than to whole papers. E0 denoted expression association; E1, nuclear enrichment or colocalization; E2, physical interaction or component mapping; E3, a perturbation-dependent change in assembly or RNA processing; E4, mechanistic reinforcement by rescue, domain mapping, epistasis, or an orthogonal perturbation; E5, linkage to an in vivo or human phenotype; and E6, target engagement together with molecular and phenotypic rescue. A study could therefore reach different tiers for different claims. The tier used in a heading, table, figure, or conclusion was not allowed to exceed the evidence described in the main text.

S1.5. Source and publication-status verification

Central, disputed, quantitative, or direction-sensitive claims were checked against the primary source at the level available for inspection. The audit recorded the model, perturbation, comparator, actual readout, direction of the result, and limits on the conclusion separately from the authors' interpretation. Bibliographic identity, DOI, final publication status, and linked correction notices were checked through PubMed and publisher records. Claims were not strengthened beyond the experiment or source version inspected.

Three retained articles had correction notices relevant to publication integrity (Table S2). The corrected items involved a funding statement, graphical-abstract labels, or the replacement of duplicated images in one figure panel. None changed the mechanistic claims for which the articles were cited in the review. No retained central source was identified as retracted or subject to an expression of concern in the records checked through 22 October 2026.

Table S2. Publication-status checks for corrected articles retained in the review.

Main-text reference	Status item	Scope of correction	Treatment in this review
[24] Salsi et al., 2025	Correction, 2026; doi:10.1093/nar/gkag798	Funding statement	Retained; mechanistic claim unchanged
[26] Kang et al., 2026	Correction, 2026; doi:10.1093/nar/gkag912	Graphical-abstract promoter labels	Retained; claim based on primary data and unchanged
[28] Yu et al., 2017	Author Correction, 2025; doi:10.1038/s41467-025-64348-4	Figure 2e KO images duplicated from WT samples; authentic KO images provided in the correction	Retained with correction disclosed; conclusion unchanged

S1.6. Conflicting evidence and bounded absence statements

Conflicting results were not reconciled by narrative averaging. For MALAT1, studies were compared by species, cell source, cell density, differentiation stage, acute versus constitutive perturbation, perturbation duration, sampling time, endpoint, and potential developmental compensation. Differences present across studies were treated as candidate moderators unless they had been isolated experimentally. Negative results were interpreted in relation to assay sensitivity, observation window, and the specific model tested.

Statements about missing evidence were bounded to the searched source set and date. The review therefore uses formulations such as 'among the primary muscle studies evaluated here' or 'in the searched source set' rather than asserting that no study exists. The statement concerning the absence of a direct MALAT1-to-speckle-to-muscle-splicing causal chain is limited to the literature identified through 20 October 2026.

S1.7. Methodological limitations

Because this was a critical narrative review, the process did not include prospective registration, duplicate independent screening, a PRISMA flow diagram, or meta-analysis. The concept-block search and citation chaining were designed to support mechanistic adjudication, not to establish an exhaustive publication count. The E0-E6 ladder is an interpretive framework developed for this review and should not be treated as a validated risk-of-bias instrument. The phase III HARBOR result was available as an official sponsor report at the search cutoff; it was used only for the reported primary-endpoint status and not as a substitute for a peer-reviewed full dataset. Reference numbers in this supplement correspond to the reference list in the main manuscript.

S1.8. Use of AI-assisted tools and figure preparation

During the preparation of this manuscript, the authors used OpenAI ChatGPT (GPT-Sol 5.6, OpenAI, San Francisco, CA, USA) for two purposes. First, it was used to prepare preliminary schematics for Figures 1–4 from scientific content specified by the authors. These schematics served only as layout drafts. All four final figures were subsequently redrawn in BioRender (BioRender.com), and a BioRender publication license was obtained for each figure. Second, it was used to check language, structure, citation placement, and internal consistency. The authors reviewed and edited all output, verified every figure label, molecular relationship, and interpretation against the cited sources, and take full responsibility for the content of this publication.
